# Conditioning and PPP processing of smartphone GNSS measurements in realistic environments

**DOI:** 10.1186/s43020-021-00042-2

**Published:** 2021-04-19

**Authors:** Ganga Shinghal, Sunil Bisnath

**Affiliations:** Department of Earth and Space Science and Engineering, Lassonde School of Engineering, York University, Toronto, Canada

**Keywords:** PPP, Smartphone, Realistic environment, Prediction, C/N_0_-based stochastic modeling, Internal phone solution, Positioning solution comparison

## Abstract

Smartphones typically compute position using duty-cycled Global Navigation Satellite System (GNSS) L1 code measurements and Single Point Positioning (SPP) processing with the aid of cellular and other measurements. This internal positioning solution has an accuracy of several tens to hundreds of meters in realistic environments (handheld, vehicle dashboard, suburban, urban forested, etc.). With the advent of multi-constellation, dual-frequency GNSS chips in smartphones, along with the ability to extract raw code and carrier-phase measurements, it is possible to use Precise Point Positioning (PPP) to improve positioning without any additional equipment. This research analyses GNSS measurement quality parameters from a Xiaomi MI 8 dual-frequency smartphone in varied, realistic environments. In such environments, the system suffers from frequent phase loss-of-lock leading to data gaps. The smartphone measurements have low and irregular carrier-to-noise (C/N_0_) density ratio and high multipath, which leads to poor or no positioning solution. These problems are addressed by implementing a prediction technique for data gaps and a C/N_0_-based stochastic model for assigning realistic a priori weights to the observables in the PPP processing engine. Using these conditioning techniques, there is a 64% decrease in the horizontal positioning Root Mean Square (RMS) error and 100% positioning solution availability in sub-urban environments tested. The horizontal and 3D RMS were 20 cm and 30 cm respectively in a static open-sky environment and the horizontal RMS for the realistic kinematic scenario was 7 m with the phone on the dashboard of the car, using the SwiftNav Piksi Real-Time Kinematic (RTK) solution as reference. The PPP solution, computed using the YorkU PPP engine, also had a 5–10% percentage point more availability than the RTK solution, computed using RTKLIB software, since missing measurements in the logged file cause epoch rejection and a non-continuous solution, a problem which is solved by prediction for the PPP solution. The internal unaided positioning solution of the phone obtained from the logged NMEA (The National Marine Electronics Association) file was computed using point positioning with the aid of measurements from internal sensors. The PPP solution was 80% more accurate than the internal solution which had periodic drifts due to non-continuous computation of solution.

## Introduction

Global Navigation Satellite System (GNSS) based positioning in smartphones has been used for personal and vehicle navigation and is now being expanded to augmented reality-based gaming, tourism applications, contact tracing, bicycle rentals, etc. Most cellphones and smartphones generally had extremely low-cost Global Positioning System (GPS) single-frequency chips tracking GPS L1 C/A-code measurements with low-cost antennas. Smartphone GNSS chipsets output position-velocity–time and limited satellite information, such as the elevation and azimuth (Guo et al. 2020). The positioning solution offered by the phone typically reached 2–3 m and degraded to tens or hundreds of meters in high noise and multipath environments. In 2016, Google introduced the availability of raw GNSS measurements for smartphones with Android N and subsequent versions and permitted duty cycling (a power-saving mechanism) to be turned off, ensuring continuous tracking of raw GNSS measurements. In 2018, the world’s first dual-frequency GNSS-enabled smartphone, the Xiaomi MI 8, equipped with a Broadcom BCM47755 chipset was launched. It is capable of tracking L1/E1 and L5/E5 code and carrier-phase signals from GPS, Galileo Navigation Satellite System (Galileo) and Quasi-Zenith Satellite System (QZSS) and single-frequency measurements from GLObal NAvigation Satellite System (GLONASS) L1 code and BeiDou Navigation Satellite System (BDS) B1 code (EGSA 2018).

Precise Point Positioning (PPP) is a viable option for improving positioning availability and accuracy for smartphones, as it is a stand-alone technique that uses precise satellite orbit, clock, and other corrections to produce cm to dm-level positioning (Bisnath and Gao 2008). Most early PPP positioning experiments were limited to single-frequency and code-only testing. Gim and Kwon-dong (2017) conducted a single-frequency pseudorange positioning test using a Nexus 9 tablet, yielding 2D and 3D Root Mean Square (RMS) positioning errors of 3.05 and 3.82 m, respectively. Gill et al. (2017) used single-frequency PPP processing to achieve RMS of 37 cm and 51 cm in horizontal and vertical components, respectively, with a Nexus 9 tablet. Sikirica et al. (2017) performed a pseudorange point positioning test under a good observation environment with a Huawei P10 smartphone, and the RMS positioning errors were ~ 10 m in the north (*N*) and east (*E*) directions and ~ 20 m in the vertical. Wu et al. (2019) processed GNSS measurements from a Xiaomi MI 8 smartphone in the dual-frequency PPP mode and obtained RMS positioning errors in the *E*, *N*, and up (*U*) directions of 21.8, 4.1, and 11.0 cm, respectively. However, it took 102 min for the three-dimensional positioning error to converge to 1 m. Aggrey et al. (2020) obtained an average horizontal error of 40 cm for dual-frequency PPP processing using the Xiaomi MI 8, with a convergence time of 38 min in ideal open sky environments.

**Fig. 1:**
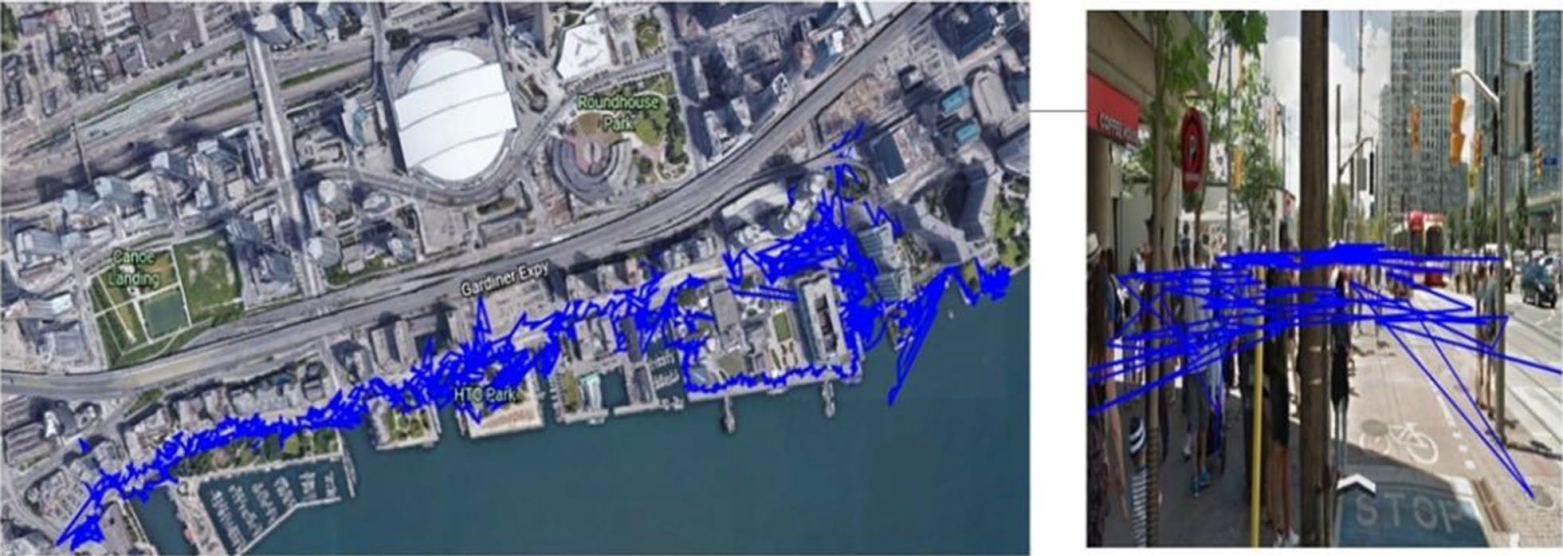
GPS L1 code-only SPP solution for a kinematic dataset collected in a high multipath, urban environment

Most GNSS smartphone positioning tests typically have been carried out in static, open-sky, ideal conditions with the phone placed flat on rooftops. These data collection methods and environments are far from those of actual phone usage, which is mostly in the kinematic mode in sub-urban and urban environments with signal blockages due to holding the phone in hand and reflections and blockages from buildings, vehicles and pedestrians. For example, Fig. 1 displays the Single Point Positioning (SPP) solution for a running pedestrian dataset collected with the phone in hand, in central Toronto, Canada, in an area characterised by tall buildings and signal blockage.

Positioning results show biases of a few meters to tens of meters, irregularity and large jumps due to multipath affecting the pseudorange measurements and the carrier-phase measurements suffering from periodic cycle slips with data gaps spanning several hundreds of seconds.

In ideal environments, accurate positioning is difficult, as smartphones possess low-cost, inverted-F linearly polarized antennas that lead to poor multipath suppression, multiple and frequent data gaps, and low, irregular signal strength. These measurement-induced errors multiply substantially in realistic environments. Secondly, smartphone measurement logging applications occasionally incorrectly compute or format measurements. Therefore, to advance positioning accuracy and availability, it is crucial to undertake a detailed analysis of measurement quality, including signal strength, multipath, measurement gaps and cycle slips in these environments and to suitably condition the measurements.

The novelty of the presented research lies in its focus on analyzing and addressing problems with smartphone GNSS measurements in realistic environments. The two major outcomes of this paper are:

1. Analyzing the quality of GNSS measurements in different multipath environments and addressing non-continuity and large errors in the PPP positioning solution due to high multipath noise and missing GNSS measurements. The use of a carrier-to-noise (C/N_0_) based stochastic model and an extrapolation-based prediction strategy is shown to reduce positioning errors and increase positioning availability.
2. Comparing the positioning accuracy and availability obtained by dual-frequency PPP processing with other techniques such as Real-Time Kinematic (RTK), SPP and the internal positioning solution for smartphones.

The paper begins with a brief description of the receivers and loggers used and various data collection scenarios. C/N_0_, multipath and data gaps are investigated. A C/N_0_-based stochastic model and a measurement prediction technique are developed and applied to the datasets. The final section of the paper compares PPP static and kinematic positioning results with SPP, RTK and smartphone internal positioning solutions.

### Raw GNSS measurement collection and analysis

A Xiaomi MI 8 phone with a BCM47755 GNSS chip was used for data collection. It tracks code and carrier-phase measurements from GPS (L1/L5), Galileo (E1/E5) and QZSS (L1/L5) and single-frequency signals from GLONASS (L1) and BDS. The SwiftNav Piksi which is a low-cost receiver was used to obtain cm-dm level reference solutions, tracking L1/L2 comparable frequency measurements for GPS, Galileo, GLONASS and BDS. The smartphone chip costs in the 10 US dollar range, while the SwiftNav Piksi chip costs a few hundred US dollars.

**Fig. 2:**
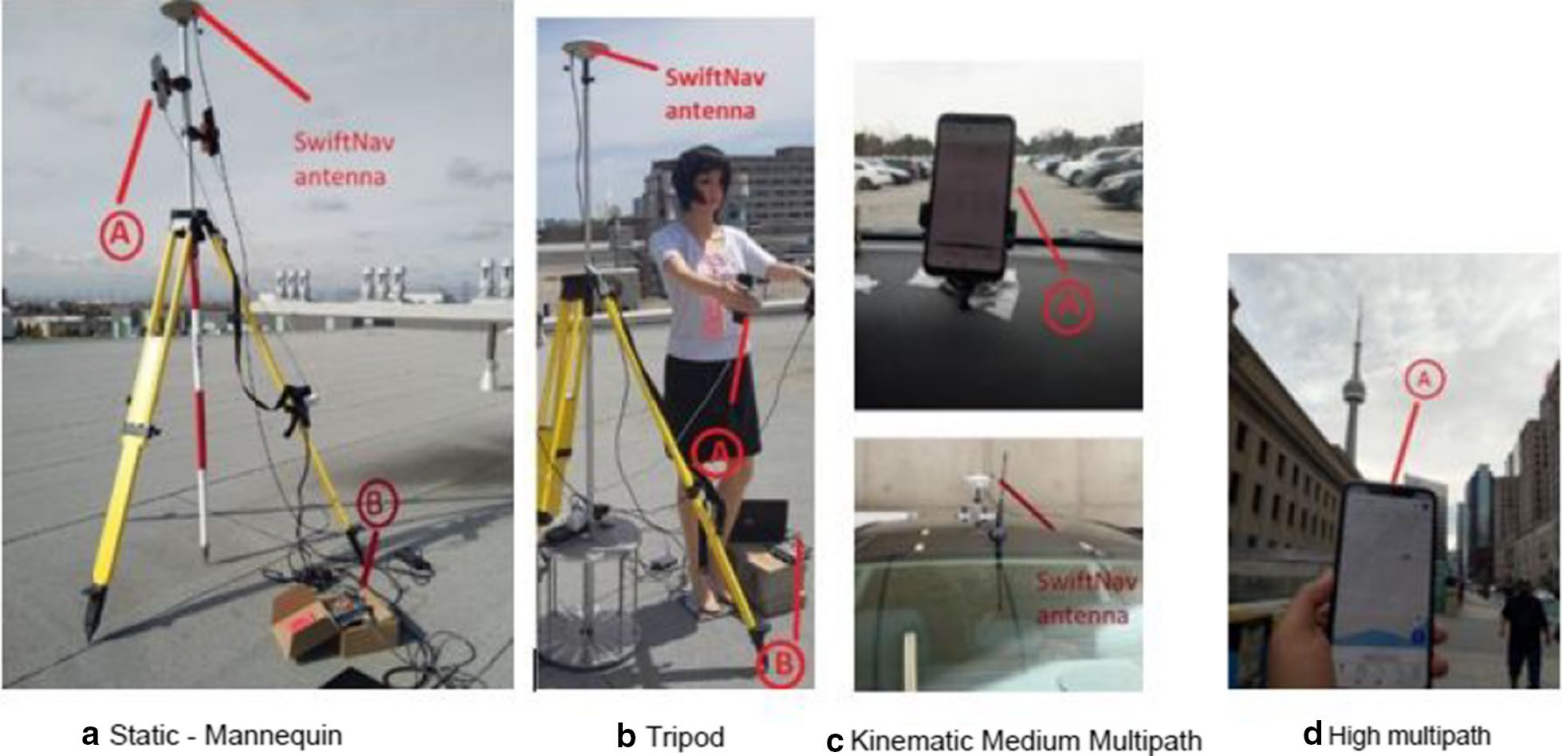
Data collection using (**a**) Xiaomi MI 8 smartphone and (**b**) SwiftNav Piksi in different multipath environments

Data were collected using the Geo + + Receiver Independent Exchange Format (RINEX) Logger (Geo++  2018) and the GNSS Logger (Diggelen and Khider 2018). Figure 2 depicts data collection using the (A) Xiaomi MI 8 smartphone and (B) SwiftNav Piksi in different multipath environments:

1. Static: Tripod-mounted phone on the rooftop for one hour on Day of Year (DOY) 225, 2019 (Fig. 2a).
2. Static: Phone taped to the hand of a mannequin, placed on a rooftop at York University, on DOY 146, 2019, for two hours (Fig. 2b).
3. Kinematic: Two datasets collected with the phone clamped to the car dashboard, driven in a medium multipath environment at York University, DOY 85 and 325, 2019. Collection duration of 41 and 33 min, respectively with SwiftNav antenna placed on the car roof (Fig. 2c).
4. Kinematic: 30-min datasets collected in a high multipath urban environment (DOY 54, 2019) and forested area (DOY 67, 2019) with the phone in hand while walking and in the pocket while skiing, respectively (Fig. 2d).

The measurements were processed with the YorkU PPP engine—a complete user PPP processor. This research focuses on dual-frequency GPS (L1 and L5) and Galileo (E1 and E5a) PPP processing in the uncombined mode. The measurements were processed using a Sequential Least-Squares (SLS) filter, as the variability in the measurement noise for smartphones GNSS measurements in different environments makes process noise tuning in Extended Kalman Filter (EKF) processing extremely challenging. An elevation angle mask of 10° and a C/N_0_ mask of 20 dB·Hz were used, as below these thresholds, measurements suffer from high multipath or have several tens of seconds of data gaps. Also, choosing an extremely high C/N_0_ mask such as 30 or 35 dB·Hz results in several satellites getting rejected when data are collected in realistic environments, further reducing the available satellite count for processing. Table 1 discusses the different PPP processing parameters deployed in the YorkU PPP processing engine. Several measurement quality parameters such as carrier-to-noise ratio, data gaps and multipath and their correlation with each other are then analysed in different multipath environments.

**Table 1:** YorkU-PPP engine processing parameters for smartphones

Processing parameters	YORK U GNSS PPP engine settings
PPP processing mode	Uncombined dual-frequency
Estimator	Sequential least squares
Antenna corrections	International GNSS Service (IGS) Antenna Exchange Format (ANTEX)
Satellite orbits and clocks	CNT-Centre National d'Etudes Spatiales (CNES)
Elevation mask	10°
C/N_0_ mask	20 dB·Hz for smartphone, 15 dB·Hz for Piksi
GNSS system	GPS, Galileo
Observations processed	L1, L5, E1, E5a
Measurement data format	RINEX 3.03
Ionospheric mitigation	Slant ionospheric delay estimation
	Using Global Ionospheric Maps (GIM’s) as pseudo-observations in the uncombined filter to mitigate and estimate the slant ionospheric error
Tropospheric modelling	Hydrostatic delay: Davis Global Pressure/Temperature (GPT)
	Wet delay: Estimated
	Mapping function: Global Mapping Function (GMF)

### Carrier-to-noise density ratio and multipath

**Fig. 3:**
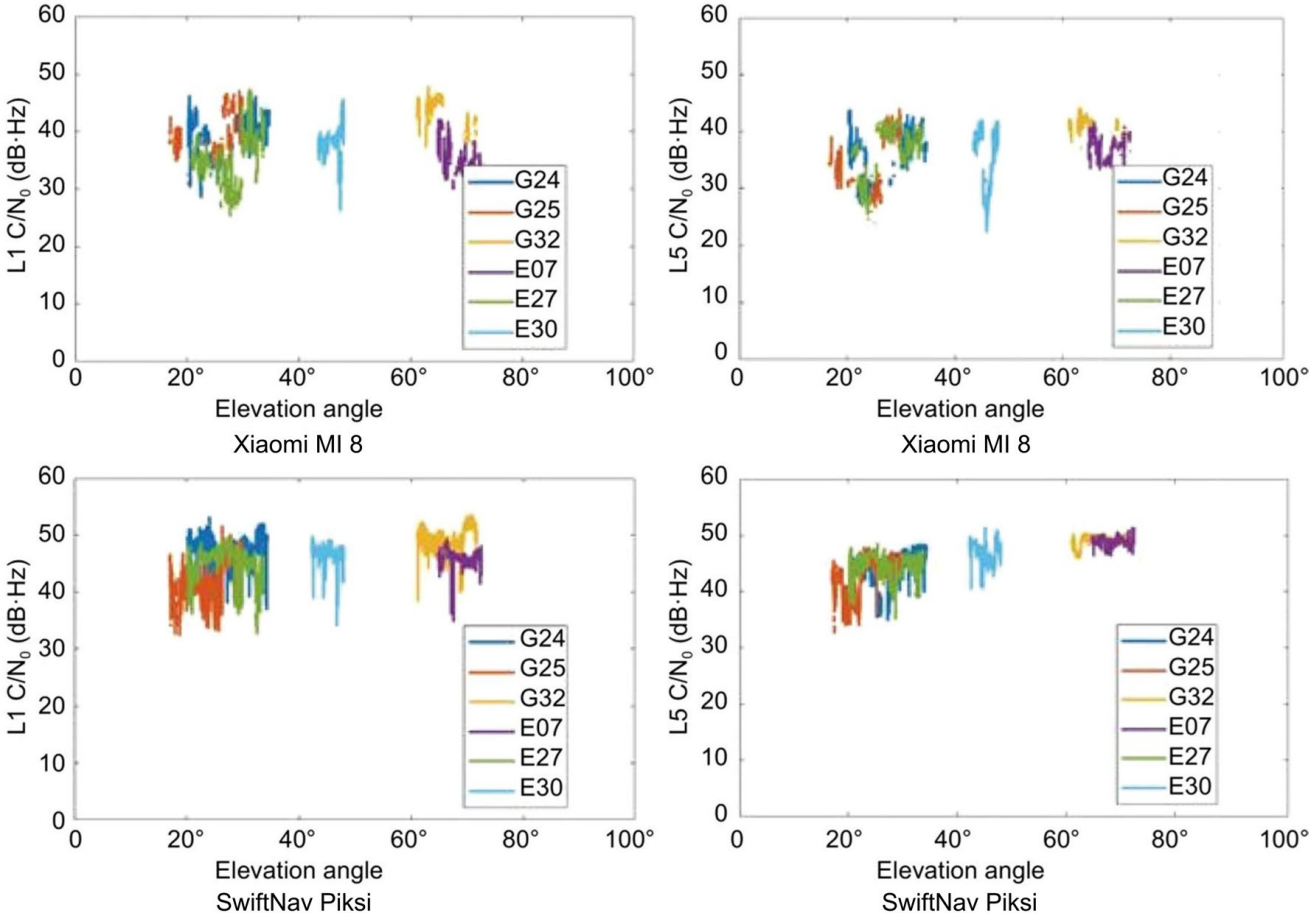
Variation of C/N_0_ with elevation angle for Xiaomi MI 8 and Piksi in a medium multipath, kinematic scenario, DOY 325, 2019

C/N_0_ is measured and outputted by the smartphone data logger and is dependent on: the power density of the incoming GNSS signal; reception area and gain of the receiver antenna; satellite elevation; and the receiving hardware, including antenna, receiver and cables (Braasch and van Dierendonck 1999; Fortunato et al. 2019). Low and irregular C/N_0_ values can be attributed to the inability of a smartphone monopole GNSS antenna to distinguish between incoming right-hand circularly polarized signals and reflected left-hand circularly polarized signals. Low signal strength and variations further compound signal multipath. The following analysis investigates these limitations in various realistic environments and their subsequent adverse effects on positioning solution quality. Figure 3 illustrates C/N_0_ as a function of the elevation angle plot for the Xiaomi MI 8 and SwiftNav Piksi in a medium multipath kinematic scenario.

The received C/N_0_ for the smartphone is not influenced by the elevation angle, while for the SwiftNav, a typical decrease in signal strength with decreasing elevation angle is observed. The duration of data collection is about 20 min and hence, there is a lack of data at all elevation angles. Due to the short time of observation for each satellite and limited number of satellites being tracked for the entire duration of data collection, there are gaps in the elevation plot. The average C/N_0_ of the smartphone L1 signal is 23% lower than that of the reference receiver. A similar performance is observed for the L5 and E5a signals. Also, for L5/E5a, it is observed that the C/N_0_ decreases above an elevation angle of 50° in medium to high multipath environments and above 60° in static open sky conditions. These findings correspond to results from Wanninger and Heßelbarth (2020) in static open sky environments.

**Fig. 4:**
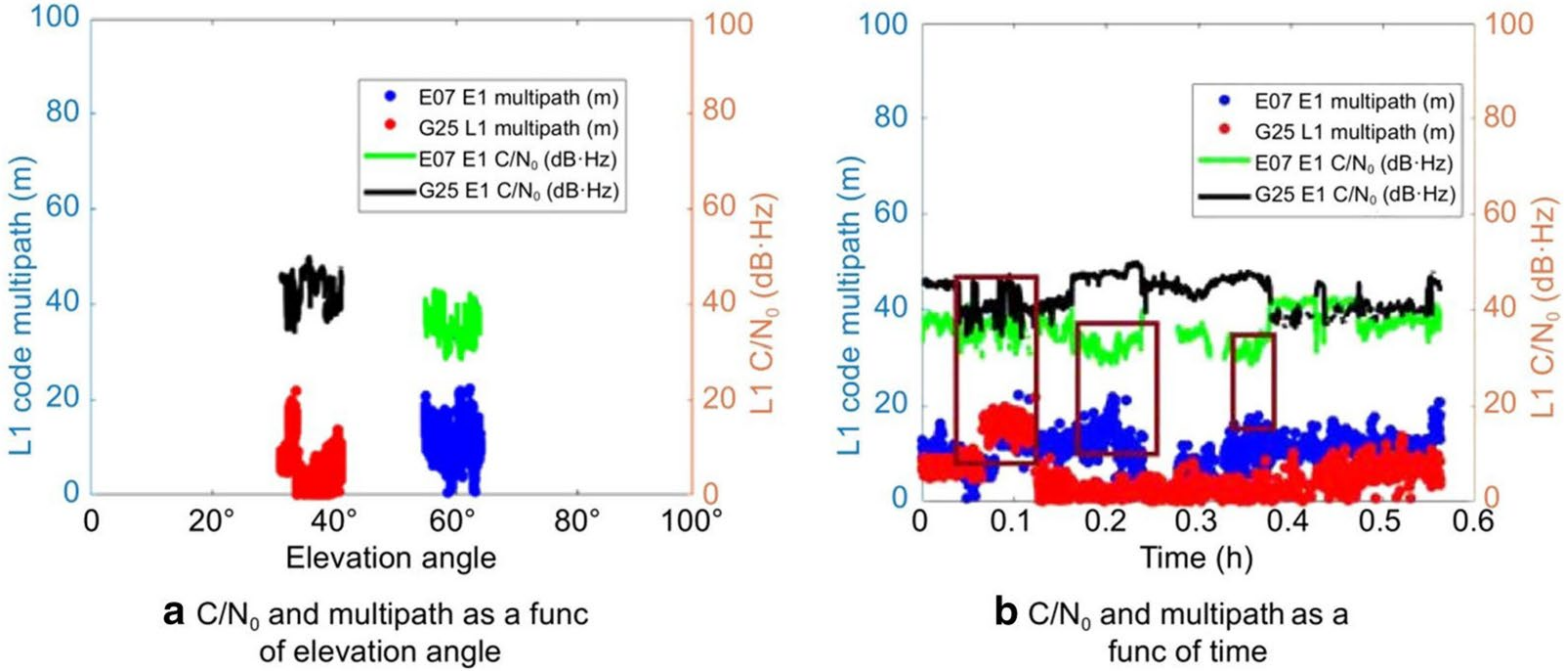
Variation in C/N_0_, elevation angle and pseudorange multipath for two satellites in A kinematic, medium multipath scenario

The pseudorange multipath was estimated by taking the linear combination of the code and carrier-phase observables with mean removed. The multipath effect on the signals for Xiaomi MI 8 is on an average 84% higher than that of the effect on the SwiftNav Piksi in a static environment and 83% higher in the kinematic environment. Since the smartphone antenna senses reflected signals from all directions, the multipath is related to the C/N_0_ rather than the elevation angle as observed in Fig. 4.

Two satellites, G25 and E07, were observed to understand the variation in multipath with a change in C/N_0_ and elevation angle since they were tracked for the entire duration of the data collection and the inferences that can be drawn are:

1. The elevation angle does not influence C/N_0_ values and multipath for smartphones as shown in Fig. 4a. G25 with a lower elevation angle of 35° as compared to 60° for E07 had a higher mean C/N_0_ of 45 dB·Hz lower L1 code multipath with an RMS of 5.9 m. E07 had a mean C/N_0_ and RMS multipath of 26.5 dB·Hz and 10.4 m, respectively.
2. There was a general decrease in multipath with an increase in C/N_0_ values as highlighted by the brown boxes in Fig. 4b as the antenna picked up reflected signals with weaker signal strength. These inferences highlight the need for a weighing model that considers these two factors.

Table 2 highlights the mean C/N_0_ and RMS multipath values for the L1/E1 and the L5/E5a signals for the smartphone in different multipath environments.

**Table 2:** Mean C/N_0_ and RMS pseudorange multipath for L1/E1 and L5/E5a frequencies for Xiaomi MI 8 in different multipath scenarios

Different multipath scenarios	Mean C/N_0_ for different frequency		RMS for different frequency	
	Mean L1/E1 C/N_0_ (dB·Hz)	Mean L5/E5a C/N_0_ (dB·Hz)	RMS L1/E1 code multipath (m)	RMS L5/E5a code multipath (m)
Low multipath	35.9	34.9	6.4	8.2
Medium multipath	35.5	33.2	10.2	16.1
High multipath	29.0	26.5	14.8	19.5

**Fig. 5:**
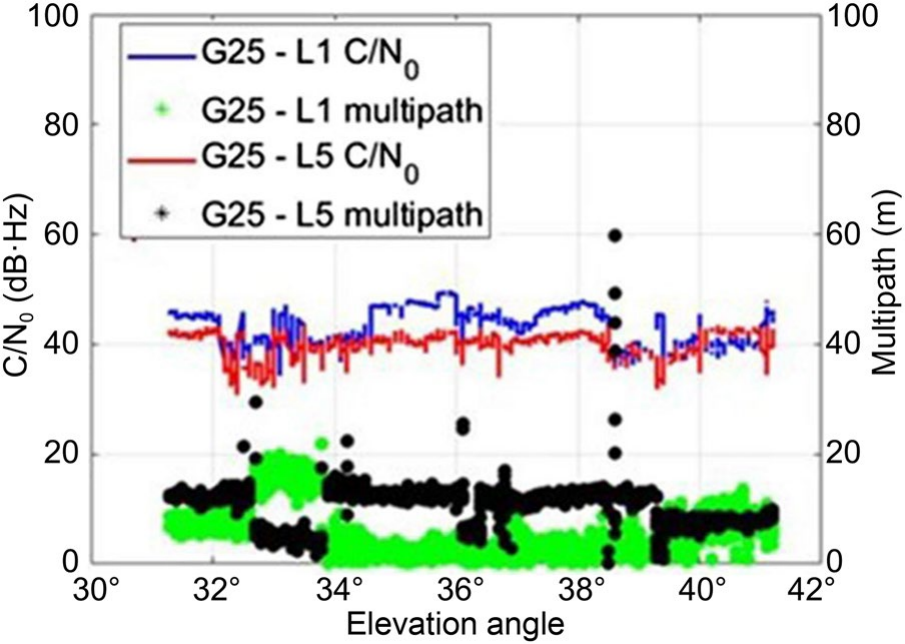
Comparison of C/N_0_ and multipath for the L1 and L5 signal for G25 for the Xiaomi MI 8

The L5/E5 signals are transmitted at higher power levels and chipping rate than L1/E1 and therefore should have higher received C/N_0_ and better noise suppression. However, the tested smartphone shows the opposite with received C/N_0_ for the L5 signal for, e.g., G25 in a medium multipath environment on an average is 3 dB·Hz lower than for L1, while the pseudorange multipath RMS for L5 is ~ 5 m more than for L1 as shown in Fig. 5.

Overall, the smartphone C/N_0_ for GPS L5 is 8% weaker than for L1, and the C/N_0_ for Galileo E5a signal is 10% less than Galileo E1 for the medium multipath, kinematic scenario as shown in Table 3. The smartphone antenna affects these signal strength and noise values as the antenna is not as sensitive for the L5/E5a signal resulting in lower signal strength. (Wanninger and Heßelbarth 2020).

**Table 3:** Mean C/N_0_ for L1 and L2/L5 frequencies for GPS and E1 and E5a/E5b frequencies for Galileo for Xiaomi MI 8 and SwiftNav Piksi in medium multipath, kinematic scenario

Smartphone names	Frequency	GPS-mean C/N_0_ (dB·Hz)	Galileo—mean C/N_0_ (dB·Hz)
Xiaomi MI 8	L1/E1	39.2	35.4
	L5/E5a	36.1	32.0
SwiftNav Piksi	L1/E1	47.4	48.1
	L2/E5b	47.9	49.1

### Cycle slips and data gaps

**Fig. 6:**
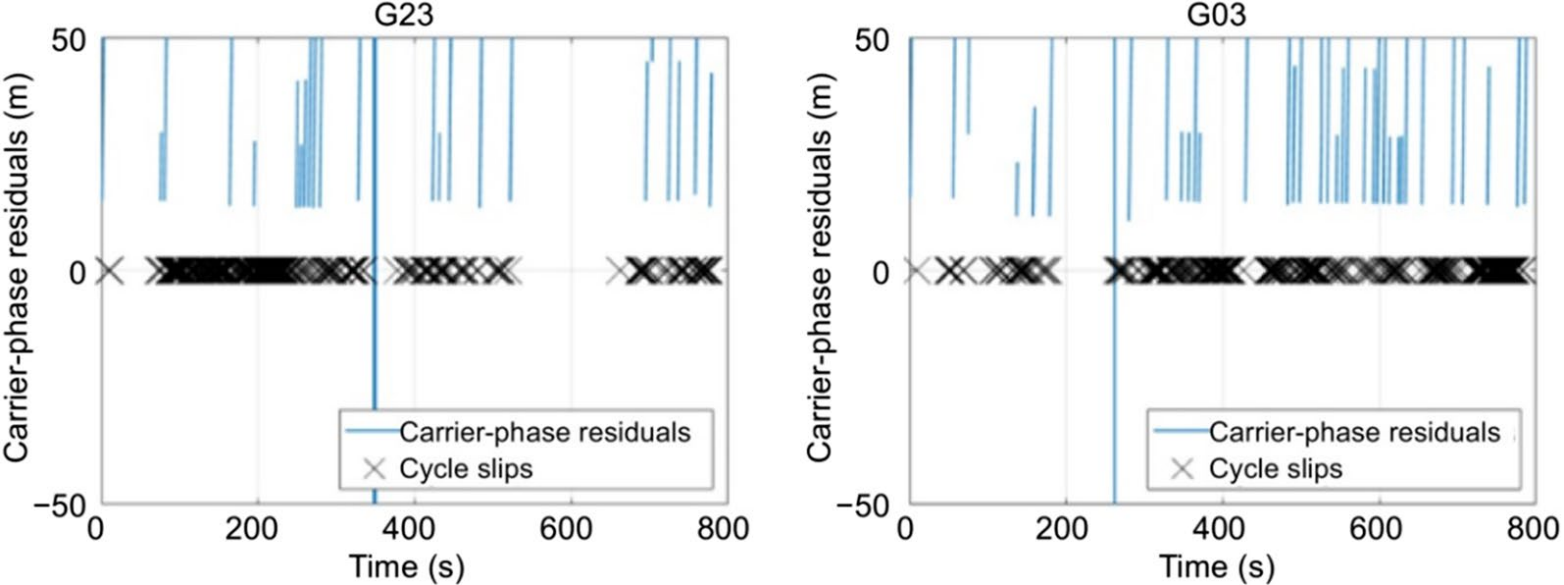
Carrier-phase residuals showing cycle slips for GPS L1 carrier-phase measurements in a high multipath forested environment (DOY 67, 2019)

In realistic data collection scenarios, there is considerable blockage and interference of GNSS signals, implying that the receiver loses lock and cycle slips occur. The antenna loses track of the signal and takes an average of 3–5 s before re-acquisition. In high multipath urban and forested areas, this signal re-acquisition can take tens of seconds. The carrier-phase observables are most vulnerable to these blockages (Marçal and Nunes 2016) and, hence, the carrier-phase tracking is not continuous (Sennott 1999). Figure 6 highlights the cycle slips affecting the GPS L1 carrier-phase measurements in a high multipath (forested) environment. The frequency of these cycle-slips increases with the increase in multipath, as is indicated by the increase in the number of black crosses. For urban canyons and forested areas, it is virtually impossible to process the carrier-phase measurements with large and frequent data gaps; hence, such datasets were excluded from further analysis.

**Fig. 7:**
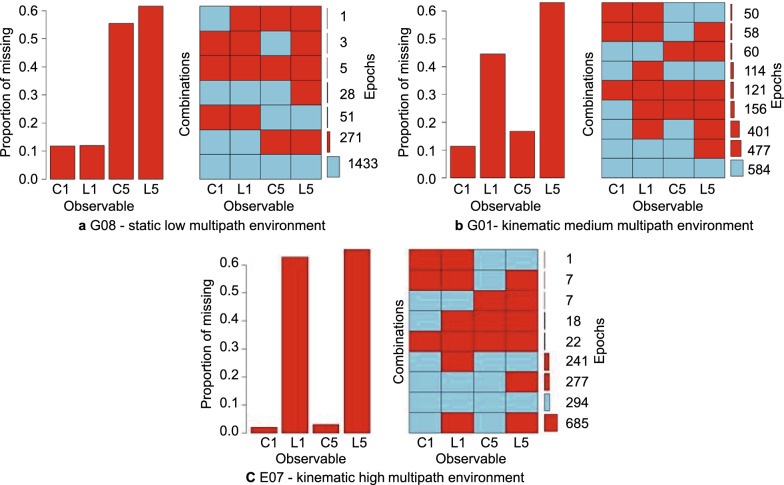
Percentage and count of missing observables (in red) for satellites in different multipath environments

Figure 7 compares the duration of the missing observables (depicted with red boxes) for two satellites in a low, medium and high multipath environment.

Each figure shows the proportion of missing measurements for any observable using a red bar graph and the different combinations of the presence and absence of the observable using the blue and red box graph. The numbers on the right indicate the duration in terms of seconds or epochs. The blue boxes signify the presence of the observable, while the red boxes indicate the absence of the observable. The L5 carrier-phase measurements are the most affected, followed by the L1 carrier-phase measurements, and the presence of all four observables decreased from 90% in low multipath static environments to just 47% in medium multipath kinematic environments and 20% in high multipath scenarios. The smartphone antenna is not as sensitive to tracking the L5 signal as compared to the L1 signal (Wanninger and Heßelbarth 2020), which explains the frequent phase-loss-of-lock and consequent data gaps affecting the L5 carrier-phase measurements the most. These data gaps in any particular observable lead to the rejection of the satellite in the processing. And subsequently, after satellite rejections due to missing observables or large residuals, the count of useable satellites falls to below the minimum requirement resulting in no solution.

**Fig. 8:**
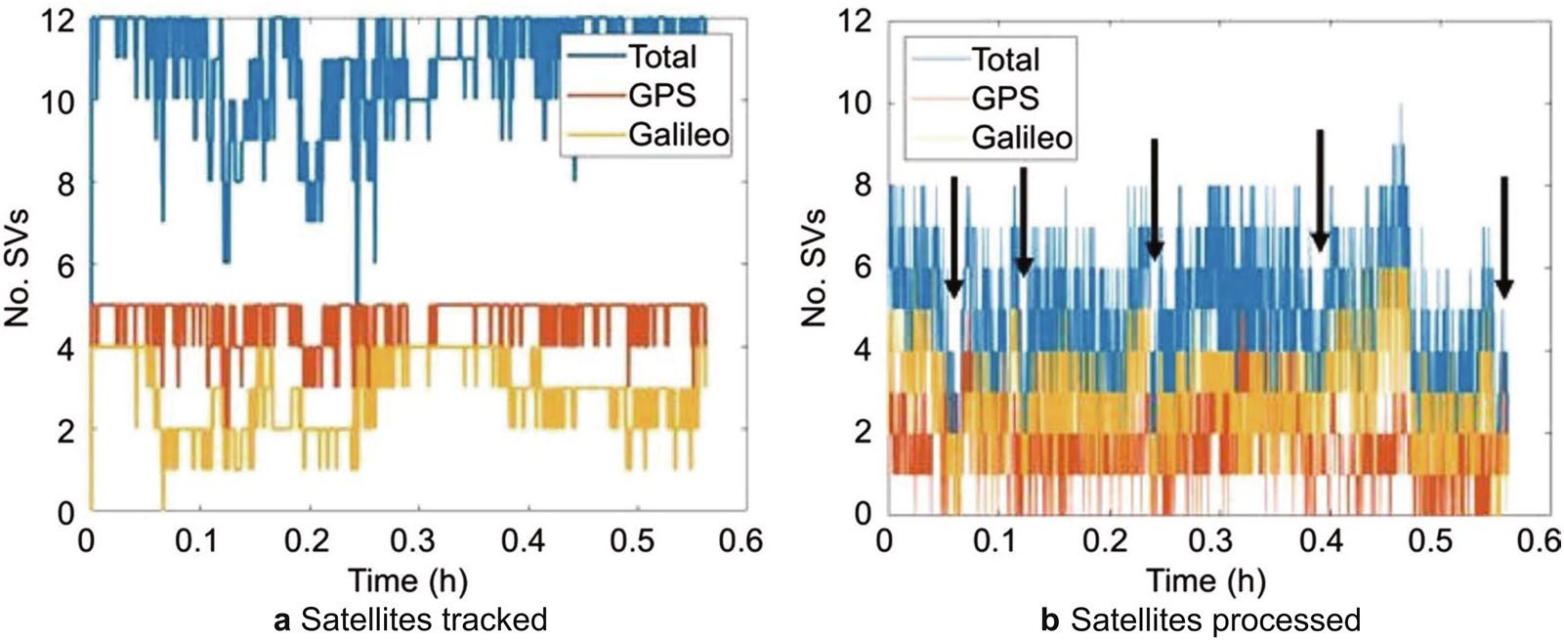
Satellites tracked versus satellites processed in a kinematic medium multipath scenario

Figure 8 compares the count of actual satellites available versus useable satellites after rejection for the driving dataset, with the solution gap portions identified with black arrows*.*

On average, 11 satellites were tracked, but only 5 could be processed after rejections due to large residual magnitude, low elevation angle or C/N_0_ value, or missing measurements. Several epochs only had 3 to 4 satellites available for processing with all 4 measurements present.

### C/N_0_-based stochastic model

Measurement weighting of parameters is another important aspect in PPP processing and since C/N_0_ is a key quality indicator in assessing smartphone GNSS measurements, this ratio can be employed in stochastic modeling (Braasch and van Dierendonck 1999). The measurement noises, ${\upsigma }_{{{\text{C}}/{\text{A}}}}^{2}$, ${\upsigma }_{{{\text{L}}1/{\text{L}}5}}^{2}$ of the C/A-code and carrier-phase measurements, respectively, at zenith is directly proportional to the square of the pseudorange chip length, $\lambda_{{{\text{C}}/{\text{A}}}}$ or carrier wavelength, and inversely proportional to the C/N_0_ (Braasch and van Dierendonck 1999). Unlike geodetic receivers, an elevation angle-based weighing strategy is not justified for smartphone GNSS measurements, as smartphones receive signals from all directions and have varying orientations due to use. (Paziewski et al. 2019; Banville et al. 2019). A C/N_0_-based stochastic model had been suggested by Banville et al. (2019) where the parameters used to compute the standard deviation of the code and carrier-phase measurements were estimated from the filter residuals. This model was adapted to assign measurement weights based on multipath noise, chipping-length or wavelength and C/N_0._ Since measurement prediction is carried out, especially for the missing carrier-phase measurements, the prediction error (1 m) was incorporated into this weighing factor. The C/N_0_-based stochastic model is as follows (Banville et al. 2019):

1 $$\sigma = a + b \times 10^{{ - \frac{1}{2} \times \frac{{{\text{C}}/{\text{N}}_{0} }}{10}}}$$

where *a* is the RMS of pseudorange multipath noise for code measurements, while it is limited to the 1 m wavelength for carrier-phase (compensated for prediction error); *b* is the pseudorange chipping length of C/A-code (293 m for L1 measurements and 29.3 m for L5 code measurements) or carrier-phase wavelength for code and carrier parameters, respectively.

### Static scenario assessment

**Fig. 9:**
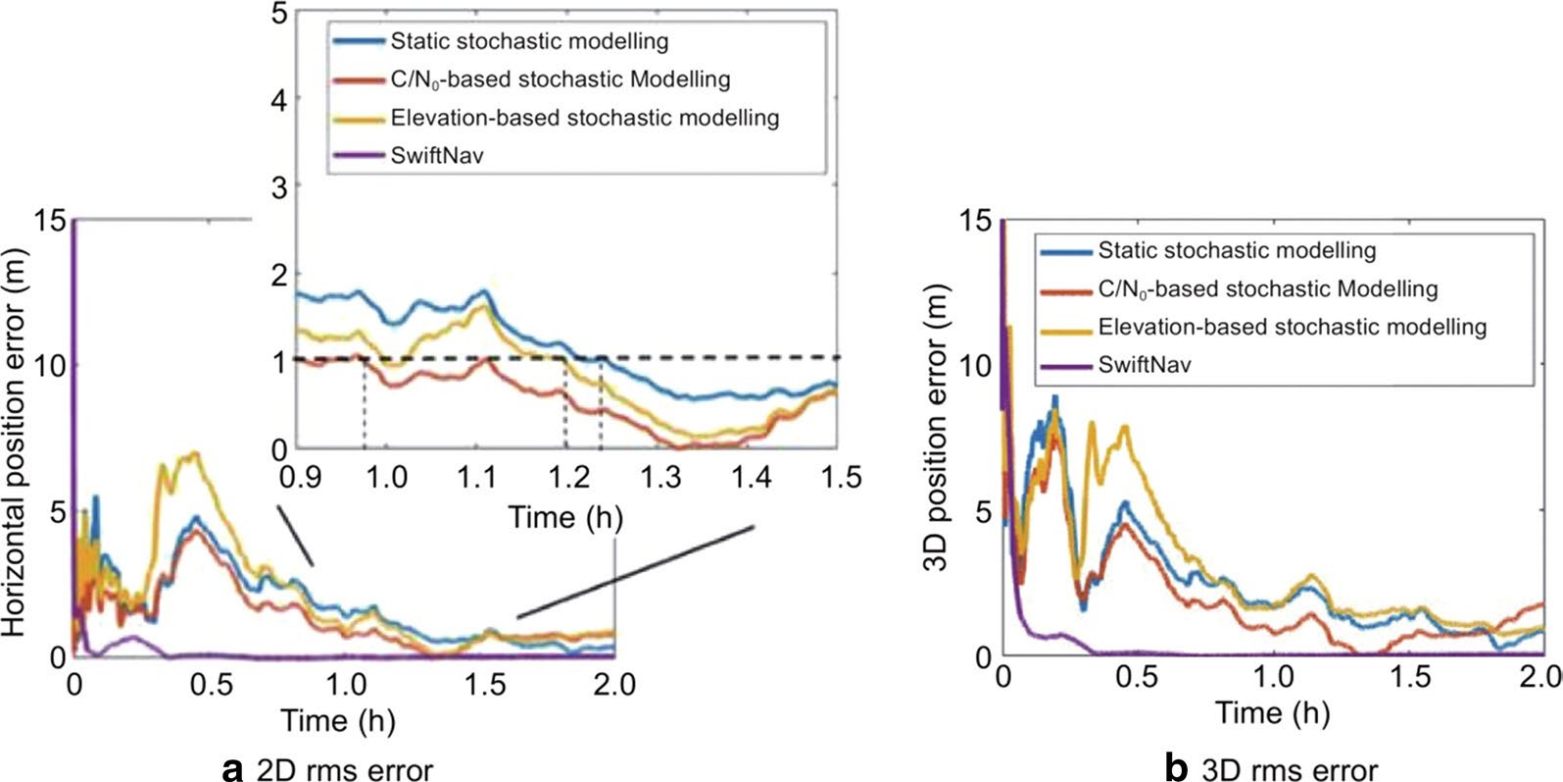
PPP 2D and 3D RMS positioning accuracy using three different stochastic models for Xiaomi MI 8 and SwiftNav Piksi measurements, DOY 146, 2019

Figure 9 compares the 2D and 3D RMS positioning accuracy and convergence time for the static mannequin dataset after employing three different measurements weighting assignments: static, elevation-based and C/N_0_-based.

The horizontal and vertical positioning accuracy after convergence is presented in Table 4.

**Table 4:** PPP positioning accuracy using three different stochastic models for Xiaomi MI 8 and SwiftNav Piksi measurements, DOY 146, 2019

Scenario	Positioning accuracy		Convergence time (min)
	Horizontal (m)	3D (m)	
Static stochastic modeling—Xiaomi MI 8	0.81	1.35	75
C/N_0_-based stochastic modeling—Xiaomi MI 8	0.72	1.27	58
Elevation-based stochastic modeling—Xiaomi MI 8	0.94	1.74	72
Elevation-based stochastic modeling—Piksi	0.14	0.16	9

To obtain sub-metre level positioning accuracy, the convergence threshold was chosen to be 1 m, and with C/N_0_-based stochastic modeling, the dataset positioning converges in 58 min, as compared to 72 min for the elevation-based model and 75 min for the static stochastic model. The C/N_0_-based stochastically modeled data show improved initialization, convergence and least 2D and 3D RMS error.

**Fig. 10:**
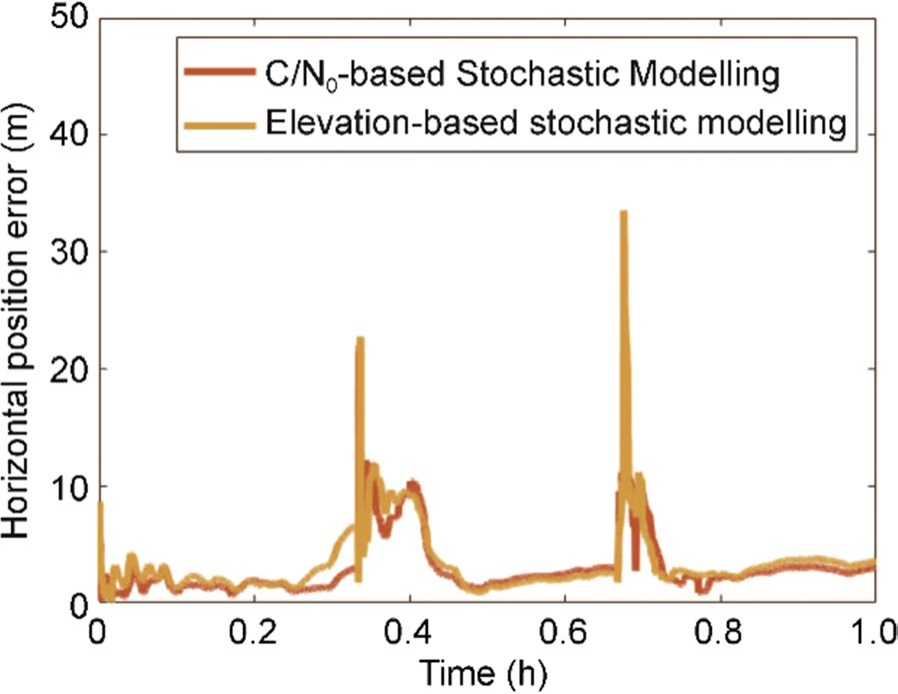
PPP horizontal positioning accuracy using different stochastic models for smartphone reset experiment

To validate the reproducibility of the results and the effectiveness of the C/N_0_-based technique over the elevation-based weighting technique, the first hour of data processing for the mannequin dataset was divided into segments of 20 min each. Therefore, the PPP processor resets every 20 min and processes the data using the C/N_0_ and elevation-based weighting strategy. The horizontal RMS of the two solutions is compared and depicted in Fig. 10.

The RMS horizontal errors for the 20-min segment for two weighting strategies are highlighted in Table 5.

**Table 5:** PPP 2D RMS positioning accuracy using different stochastic models for smartphone reset experiment, DOY 146, 2019

Time Interval (min)	2D RMS error (m) C/N_0_-based weighting	2D RMS error (m) Elevation-based weighting	Percentage difference (%)
0–20	1.7	2.8	64.7
20–40	5.2	5.3	1.9
40–60	4.1	5.1	24.4

These results depict the effectiveness of a C/N_0_-based technique due to significantly reduced initialization error on each reset and lower RMS error. Overall, the mean RMS error was 30% lower for the C/N_0_-based weighting strategy for the three segments.

The post-fit residuals for the three weighing models are compared in Table 6.

**Table 6:** Post-fit residual RMS for different stochastic models for Xiaomi MI 8, DOY 146, 2019

Scenario	Post-fit C1 (m)	Post-fit L1 (cm)	Post-fit C5 (m)	Post-fit L5 (cm)
Static stochastic model	13.8	32.7	2.3	25.8
C/N_0_-based stochastic model	4.3	6.0	1.9	4.3
Elevation-based stochastic model	5.0	9.0	2.1	6.1

**Fig. 11:**
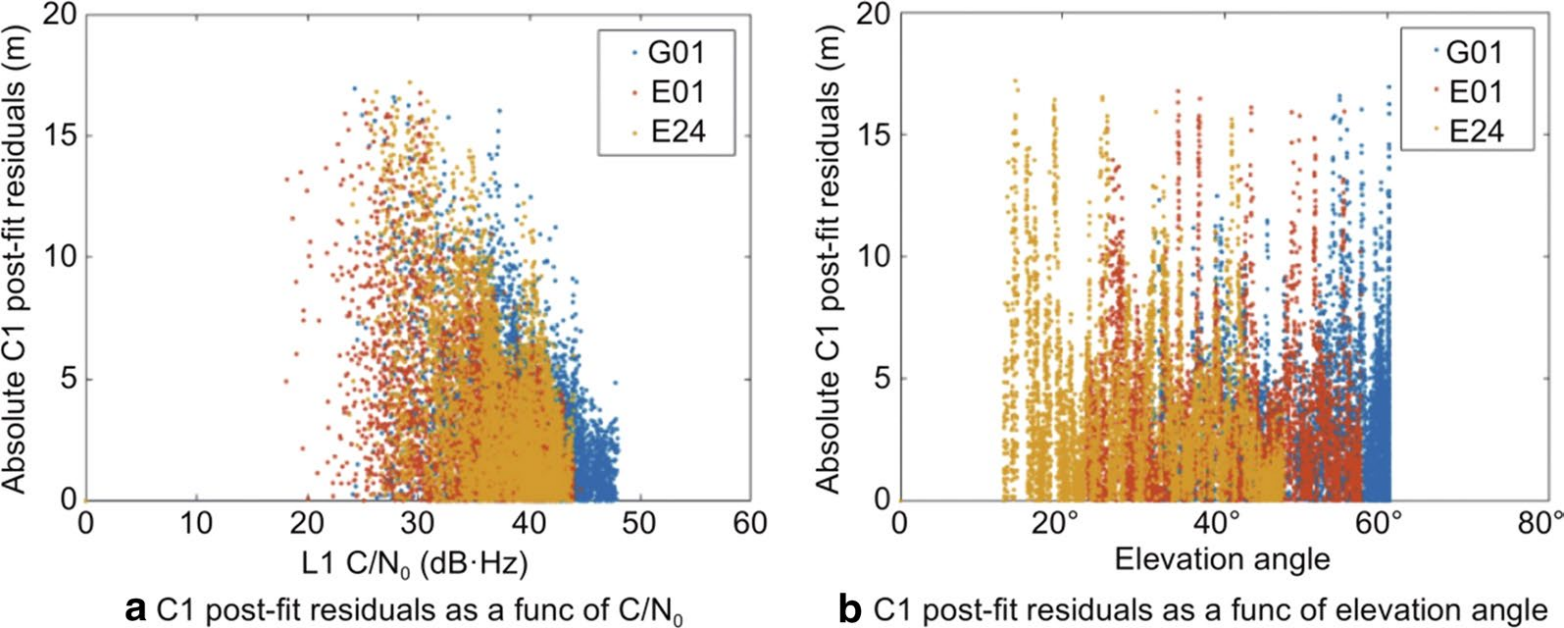
Variation of PPP C1 post-fit residuals with C/N_0_ and elevation angle for Xiaomi MI 8, DOY 146, 2019

The C/N_0_-based model outperformed the other two models in terms of residual magnitude, as there is a decrease in residual magnitude with increasing C/N_0_ values. No such dependence can be observed for the residuals and the elevation angle as seen in Fig. 11, where the C1 post-fit residuals for three satellites have plotted against C/N_0_ and elevation angle.

### Measurement prediction

**Fig. 12:**
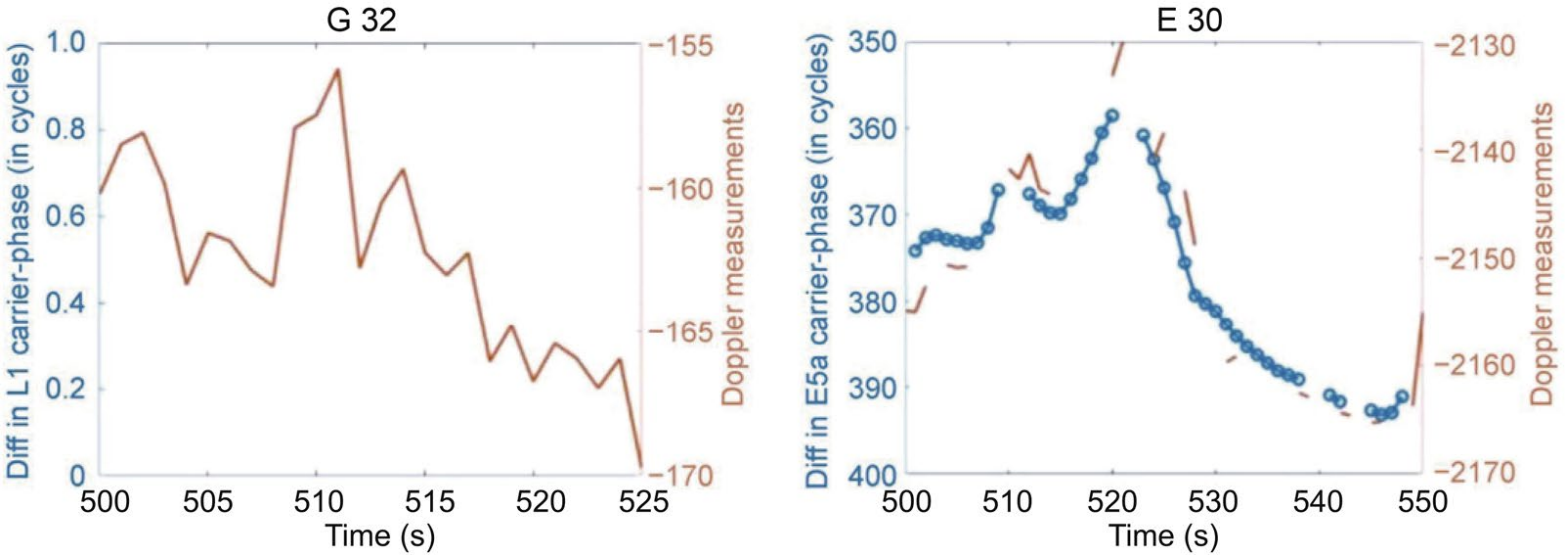
Change in carrier-phase measurements compared to Doppler measurements for a kinematic dataset in a medium multipath environment, DOY 325, 2019

A measurement prediction technique has been devised to predict missing measurements to increase positioning solution availability. As mentioned earlier, it was extremely difficult to tune the noise parameters for the highly variable smartphone raw GNSS measurements in realistic environments in an EKF filter. Accordingly, the EKF filter could not used for measurement prediction. Hence, a separate measurement prediction technique had to be devised. Various real-time extrapolation and estimated Doppler prediction techniques were tested; however, they were discarded for a simple linear extrapolator, as it provides lower prediction error for filling data-gaps in low to medium multipath environments. For example, the estimated Doppler prediction technique (Li et al. 2019) is limited by a lack of knowledge of dynamics without the aid of an Inertial Measurement Unit (IMU). The current research focuses on GNSS-only processing. The logged Doppler measurements show large variability and gaps, as can be seen in Fig. 12 and therefore cannot be used for prediction.

For satellite G32, there are no L1 carrier-phase measurements depicted by the jumps in the logged Doppler measurements, even though the satellite had a mean elevation of above 70° and a C/N_0_ value averaging 35 dB·Hz. The lack of L1 carrier-phase measurements in such a scenario could be attributed to the low-cost antenna. For satellite E30, the L5 Doppler measurements are not continuous, with more frequent and larger gaps than for the carrier-phase data gaps. The occurrence of long durations of missing Doppler measurements can be attributed to poor carrier-phase tracking in challenging environments and due to the low-cost antenna.

**Fig. 13:**
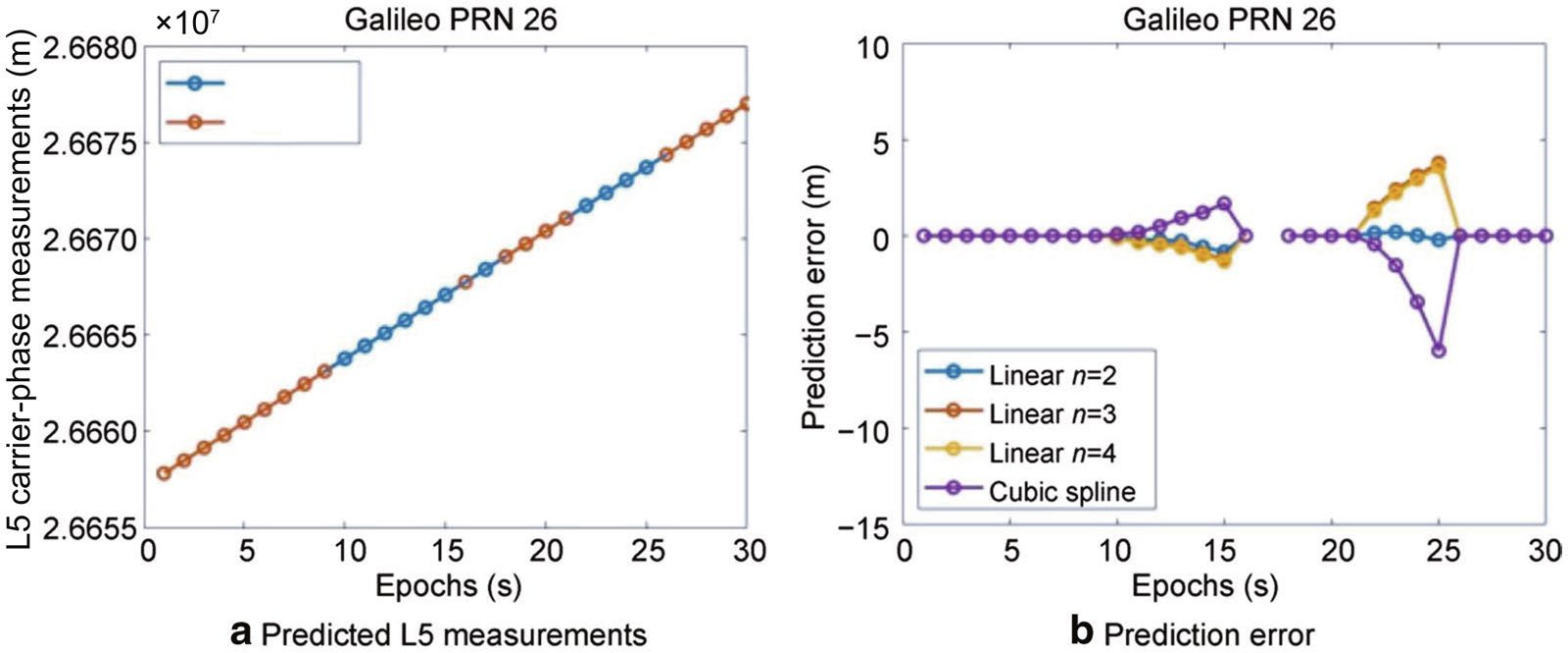
Predicted L5 measurements and prediction error using different extrapolation technique

Different extrapolations methods were tested and a summary is presented in Fig. 13 using the example of satellite G26 L5 carrier-phase measurements.

Artificial gaps ranging from 4 and 5 s were synthesized. ‘*n*’ corresponds to the number of past measurements used to predict the next measurement in case of a gap for a linear extrapolator. The linear model is a simple mathematical technique that computes the slope based on the past measurement and uses it to predict the next measurement. If the epochs are taken as *X* and the carrier-phase / pseudorange measurements are taken as *Y*, then

2 $$m = \frac{{n {\Sigma } XY - {\Sigma }X {\Sigma }Y}}{{n{\Sigma }X^{2} - \left( {{\Sigma } X} \right)^{2} }}$$

3 $$c = \frac{{ {\Sigma }Y - m{\Sigma X}}}{n}$$

where n is the number of past measurements considered. The extrapolated measurements are taken into consideration if the gap is greater than one epoch. *X* is the index to count the number of epochs; *Y* is the value of the observable being predicted. The next measurement at *X* can be predicted as:

4 $$c + m\,{\times}\,x$$

As seen in Fig. 13, the carrier-phase measurements already have a data gap present at epoch 17. The linear extrapolator with the past 2 measurements produces an RMS prediction error of 16 cm over the 4 epochs (epochs 21–24, inclusive), while the prediction error using 3 and 4 measurements is 2.8 and 2.6 m, respectively. The cubic spline extrapolation yields an error of 3.5 m for the same. Based on the analysis carried for a wide range of data, it was concluded that better prediction occurs with linear extrapolation using measurements from the previous two epochs. This result can be attributed to the large variability in the magnitude of code and carrier-phase measurements over successive epochs in a dynamic environment and hence, it is best to use measurements from a minimum number of past epochs to predict ahead. The positioning solution obtained with the application of this technique is investigated next.

### Kinematic scenario assessment

**Fig. 14:**
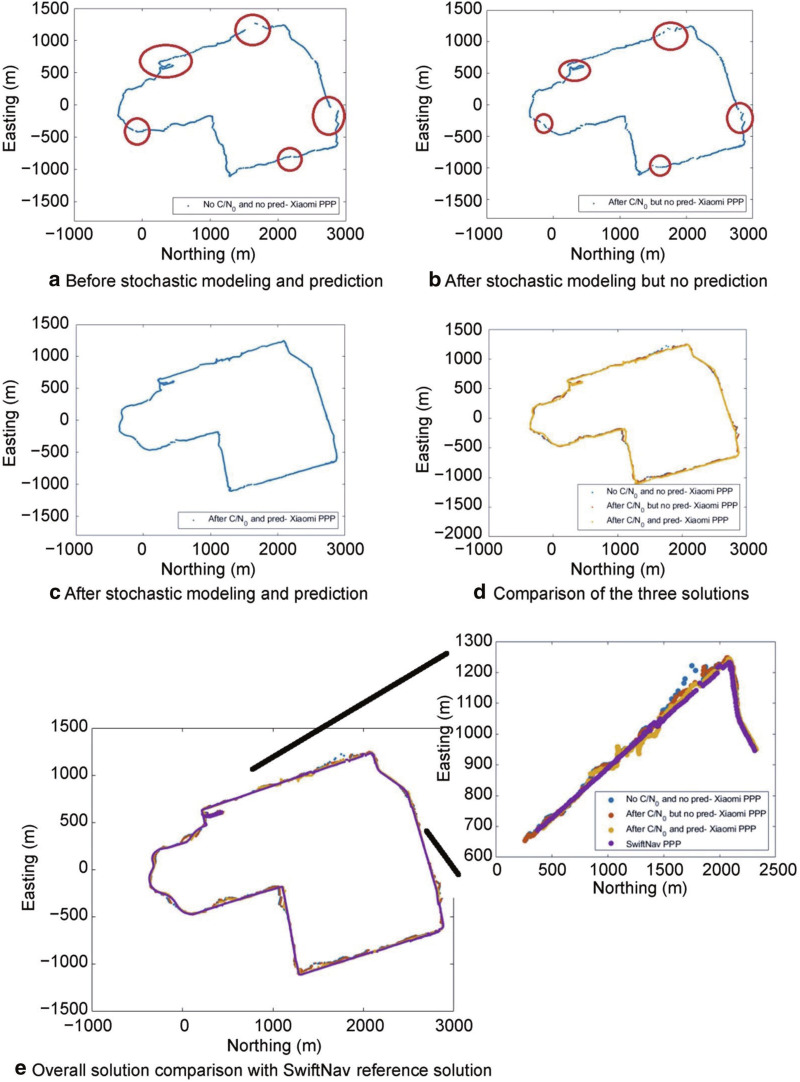
Xiaomi MI 8 PPP kinematic track before and after C/N_0_-based stochastic modeling and prediction with SwiftNav Piksi reference track, DOY 325, 2020

Suburban vehicle data collected on DOY 325, 2019 suffered from frequent data gaps. Figure 14 shows the vehicle trajectory positioning accuracy and availability before the application of the C/N_0_-based stochastic model and measurement prediction techniques (Fig. 14a), after the implementation of the C/N_0_-based stochastic model but no prediction (Fig. 14b), and after the application of the C/N_0_-based stochastic model and the prediction strategy (Fig. 14c). The red ovals indicate periods of no solution and/or solution divergence. The three solutions have been plotted together (Fig. 14d) and with the SwiftNav PPP solution (Fig. 14e).

**Fig. 15:**
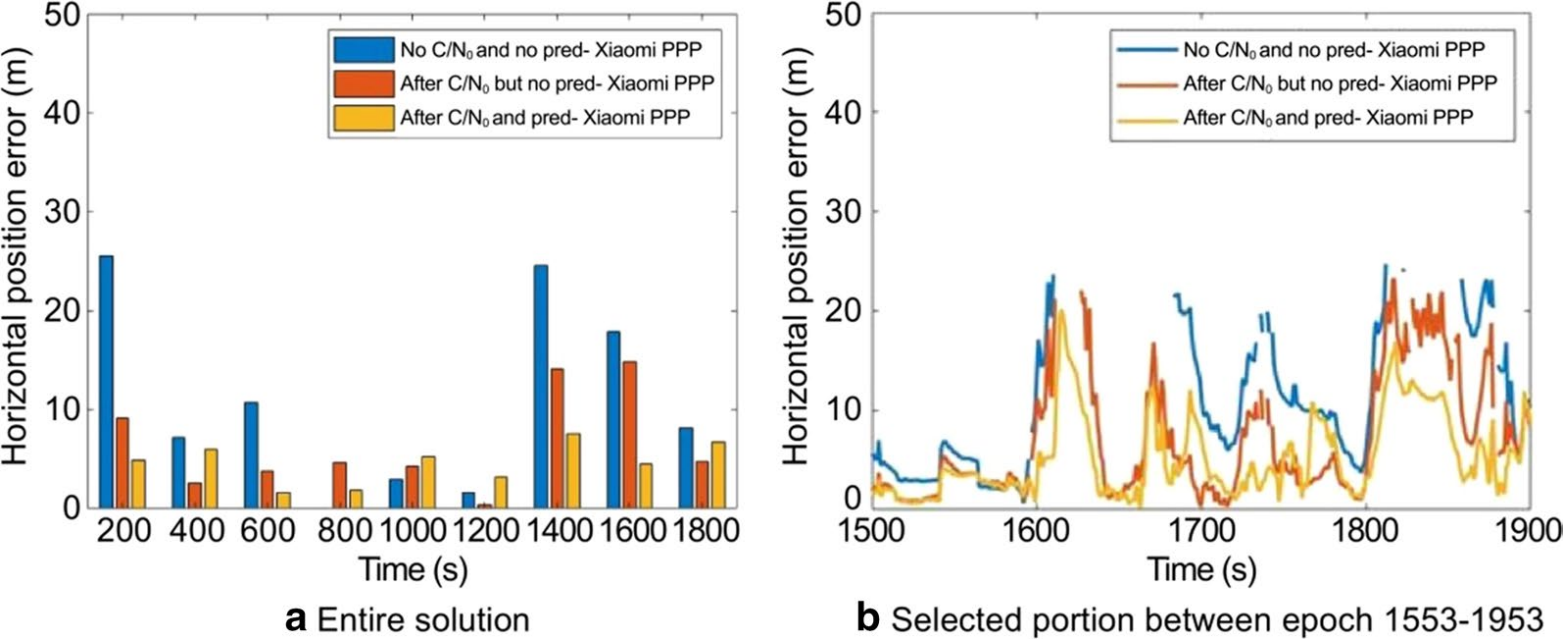
Horizontal positioning error before and after implementing the C/N_0_-based stochastic modeling and prediction for Xiaomi MI 8, DOY 325, 2019

The objective of the analysis is to compare the overall improvement in the horizontal positioning accuracy and availability of the Xiaomi PPP solution with itself before and after the implementation of the C/N_0_-based stochastic model and prediction technique using the Piksi PPP reference solution as shown in Fig. 15.

Notably, the phase centers of the SwiftNav antenna and the phone antenna were not aligned. The SwiftNav antenna on the vehicle roof had better signal availability, while the smartphone being inside the car had additional signal blockages, fewer tracked satellites, considerable missing carrier-phase measurements and multipath affecting the code measurements. Despite these adversities, with the implementation of prediction and C/N_0_-based stochastic model, a solution with 100% availability is achieved and the accuracy of the positioning solution improved, especially over the region between epoch 1553 and 1953 – highlighted in Fig. 14. Overall, there is a 64% decrease in horizontal positioning standard deviation and RMS error and a 1.3% increase in solution availability to 100% after the conditioning and prediction as shown in Table 7.

**Table 7:** RMS and STandard Deviation (STD) deviation of horizontal positioning error and availability of solution before and after implementing C/N_0_-based stochastic modeling and prediction for Xiaomi MI 8, DOY 325, 2019

Scenario	Horizontal RMS (m)	Horizontal STD (m)	Solution availability
Before prediction and C/N_0_-based stochastic model	12.7	7.6	1995/2021 = 98.7%
Before prediction but after C/N_0_-based stochastic model	8.0	5.1	2004/2021 = 99.2%
After Prediction and C/N_0_-based stochastic model	4.6	2.7	2021/2021 = 100%

For the major outage between epochs 1553–1953, 23% (91/400) of epochs has no solution before the C/N_0_-based stochastic model and prediction, which decreased to 5% (21/400) after the stochastic model implementation, while with both the conditioning and prediction, a 100% continuous solution was obtained. The RMS horizontal positioning error decreases from 15.5 m to 8.4 m and finally to 5.8 m for the portion between epochs 1553–1953.

As seen, the prediction and C/N_0_-based stochastic model improved the position availability and accuracy, considerably. The RTK solution for the same had an availability of 98% (1983/2021), suggesting the successful use of a similar prediction technique for it as well. Further, several other datasets were tested for the same and the positioning accuracy was compared against the SPP, RTK and internal positioning solution.

### Comparison of various positioning solutions

This section compares the PPP positioning solution after conditioning of raw measurements with the SPP, RTK and internal phone solutions. Both static and kinematic data have been compared and quantitative comparison results are tabulated.

### Static dataset

**Fig. 16:**
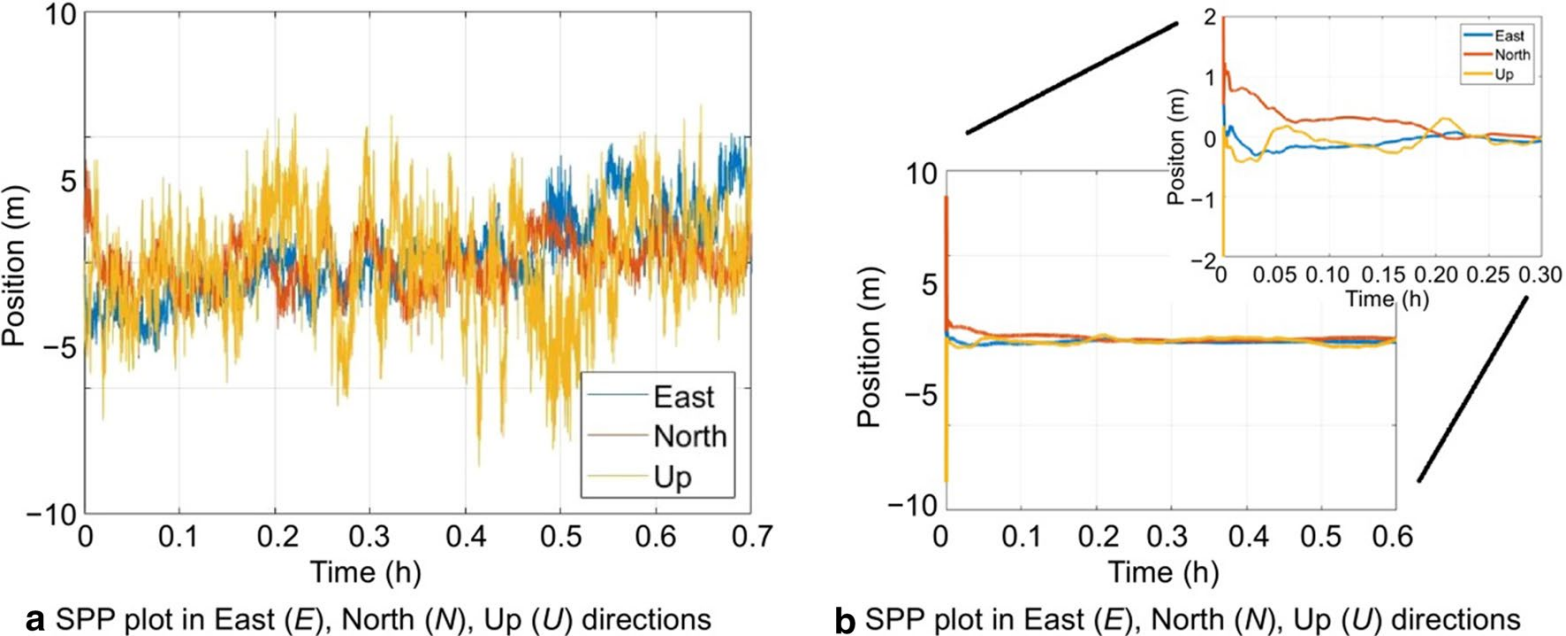
Positioning error for SPP and PPP solutions, DOY 225, 2019

Most GNSS chips compute positioning solutions using the SPP technique using the GPS L1 code measurements. Hence, it is deemed necessary to test the positioning accuracy of the SPP technique with PPP solution obtained by the YorkU PPP engine. A static dataset was chosen and a weighted, least-squares, epoch-by-epoch, GPS L1 code SPP solution was computed. Data collected on DOY 225, 2019, by attaching the phone to a tripod, was chosen since it represents the standard open sky, static positioning test. In degraded and difficult environments, the SPP solution quality was expected to deteriorate, further. Figure 16 compares the two positioning solutions where the smooth and continuous PPP solution converges to a horizontal positional accuracy of 1 m in 9 min.

The RMS in the horizontal and vertical directions are 22 cm and 35 cm, respectively, whereas the SPP solution is scattered and irregular with a horizontal and vertical RMS of 2.0 m and 3.0 m, respectively, as shown in Table 8.

**Table 8:** Position accuracy for SPP and PPP solutions, DOY 225, 2019

Position methods for Xiaomi MI 8	Position accuracy in different dimensional (m)	
	2D RMS	3D RMS
SPP	2.0	3.0
PPP	0.2	0.3

### Kinematic dataset

**Fig. 17:**
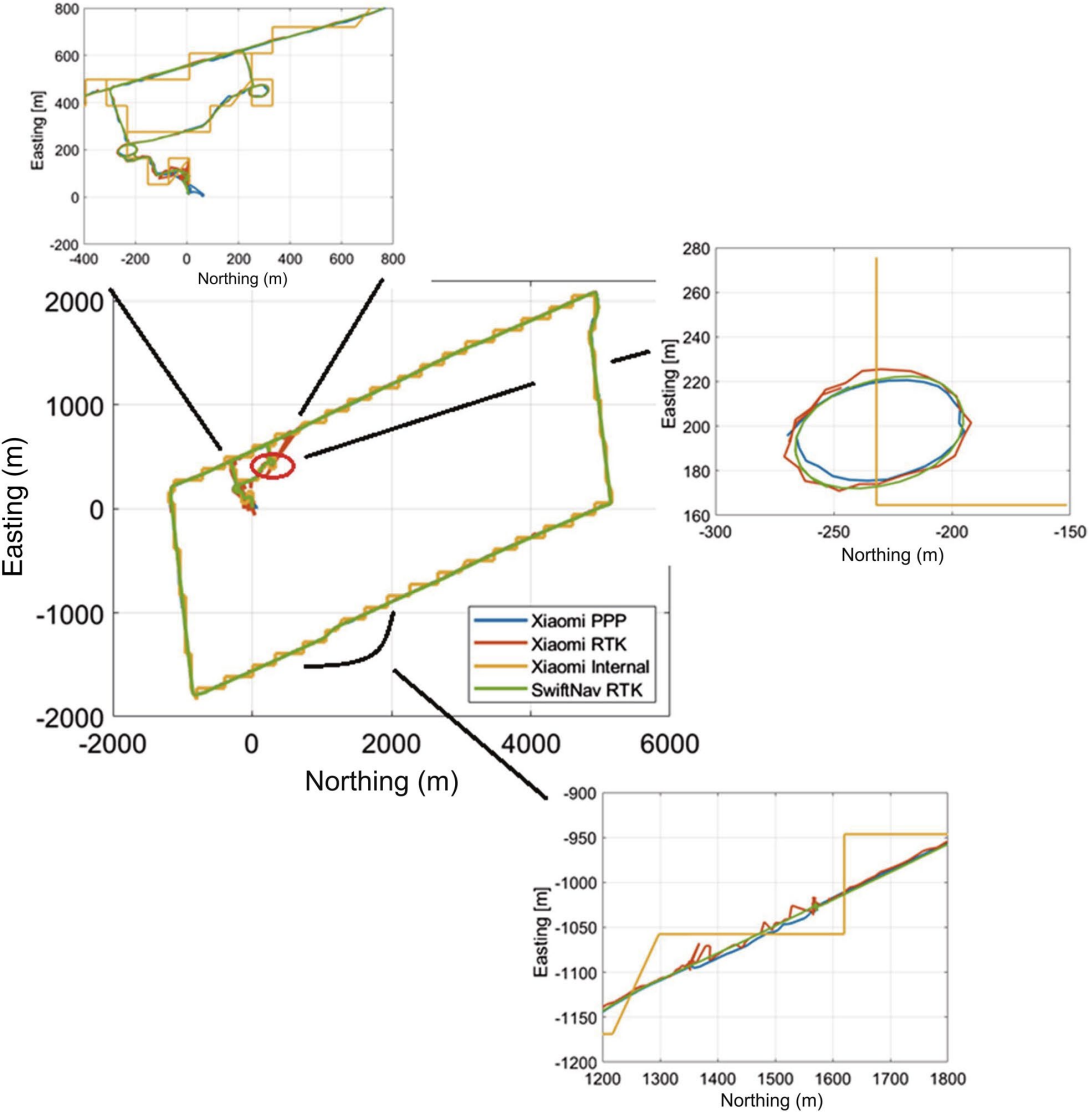
Positioning solution comparison for a kinematic dataset using different positioning techniques, DOY 85, 2019

The kinematic dataset collected on DOY 85 was processed in PPP mode after implementing the C/N_0_-based stochastic modeling and prediction technique and the solution is compared against RTK and internal smartphone solutions. It is expected that the internal solution was computed using SPP with the aid of measurements from internal sensors. Due to lack of internet connection, the solution was not aided by measurements from cell towers and hence, the positioning solution periodically showed drifts. The RTK baseline, which matched the PPP baseline, was ~ 1 km. The base station used consisted of a geodetic antenna and Topcon NET-G3A, and RTKLIB ver.2.4.2 was used to process the GPS and Galileo measurements to maintain uniformity with the smartphone PPP processing. An elevation angle mask of 10° and a C/N_0_ mask of 20 dB·Hz were maintained similar to the PPP processing. Precise orbit and clock products were used for processing along with slant ionospheric error modeling. As shown in Fig. 17, the internal solution obtained from the smartphone represents a zig-zag pattern, which possibly relates to the drift in the solution. The phone does not consistently determine or update its position using GNSS and there was no connection to cell towers. The PPP solution is far smoother, continuous and follows the reference track of the Piksi RTK solution. Ideally, over short baselines, the RTK solution is better or as good as the PPP solution. However, when the smartphone raw measurements were processed in RTKLIB, around 8% of the measurements were rejected resulting in solution outages in the presented RTK solution than the presented PPP solution as can be seen in Fig. 17.

The internal solution has periodic deviations and higher positioning error as compared to the RTK and PPP solutions, as shown by the track in yellow. The RMS and standard deviation of the horizontal positioning error are depicted in Table 9.

**Table 9:** Comparison of RMS and std of positioning error for PPP, RTK and internal positioning solution for Xiaomi MI 8, DOY 85, 2019

Processing scenario Xiaomi MI 8	Horizontal positioning error (m)		Solution availability (%)
	RMS	STD	
PPP	6.84	5.20	2443/2443 = 100
RTK	4.55	2.80	2253/2443 = 92
Internal solution	42.33	16.21	2443/2443 = 100

**Fig. 18:**
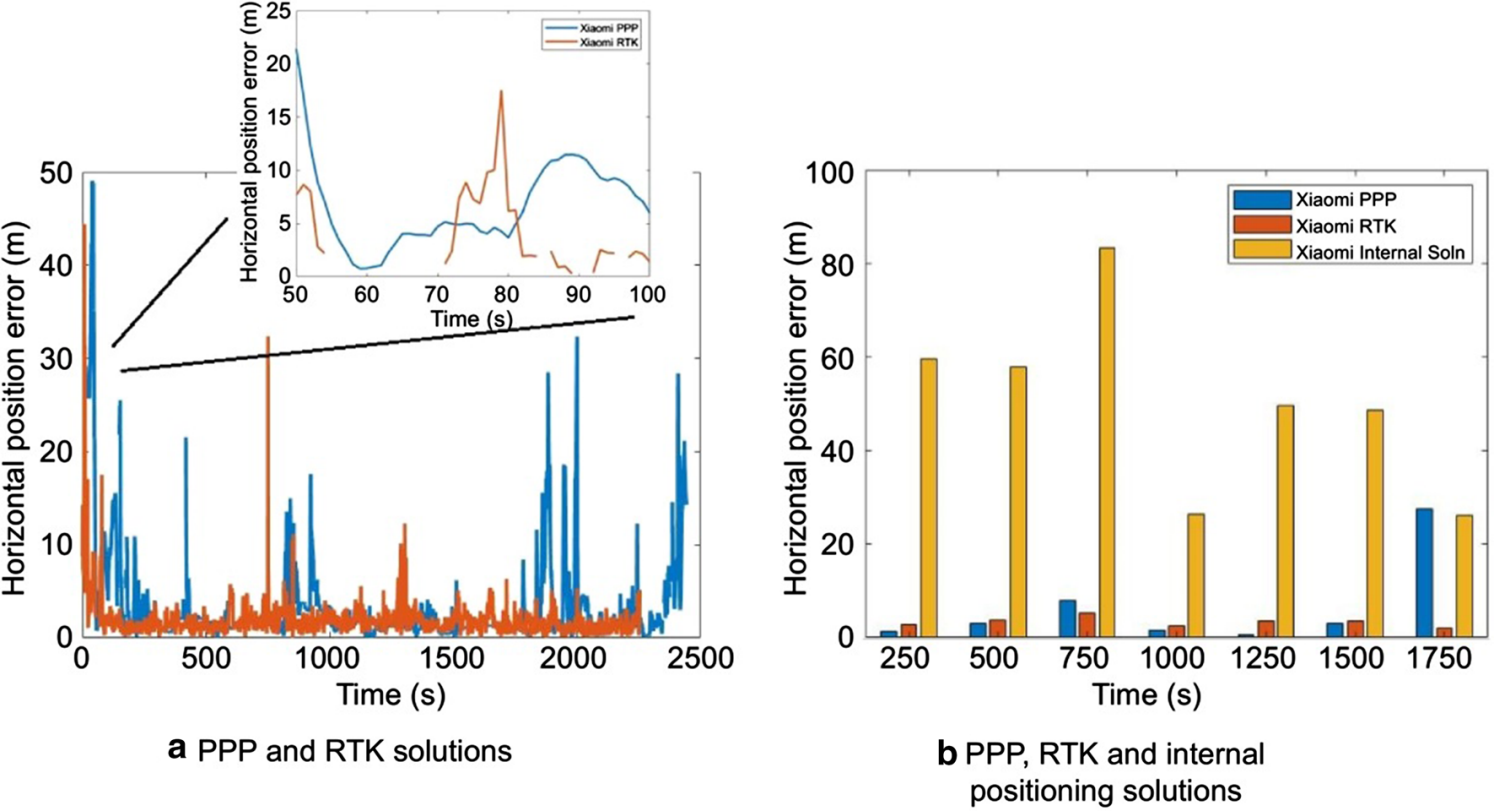
Comparison of 2D RMS error for PPP, RTK and internal positioning solution for Xiaomi MI 8, DOY 85, 2019

The first few epochs are ignored, as the car was parked in a high multipath environment (parking lot). The RTK RMS is 33% lower than the PPP solution but only have 92% solution availability, as marked by the data gaps in Fig. 17a between epochs 50–100. The gaps in the RTK solution can be attributed again to the rejection of epochs with missing measurements, a problem which is solved by prediction in the PPP processing. Figure 18b represents the positioning error comparison for the three positioning solutions. The internal solution performs the worst in the circular track, shown in Fig. 17, due to frequent switching on and off of the position tracking system. During this circular portion of the track, the PPP solution outperforms the RTK and internal solution. The internal solution failed to follow the circular track, while the RMS of the RTK solution is 6.1 m compared to the PPP solution of 5.6 m. The RTK solution over the circular portion of the track shows drifts from the reference track which is due to frequent loss of solution as the satellite count drops to below the minimum required as a result of the rejection of satellites due to missing measurements.

## Conclusions and future work

This research attempts to address gaps in GNSS research related to smartphones by focusing on analyzing and addressing challenges posed by the non-continuous and poor quality of raw measurements collected in realistic environments. The York-PPP engine has been customized for smartphone GNSS measurements through improved C/N_0_-based stochastic modeling and measurement prediction. Tested in realistic static and scenarios, positioning accuracy is compared with results from SPP processing, internal smartphone positioning solutions and RTK processing. In static testing, the PPP RMS in the horizontal direction is 22 cm and 35 cm in the up direction. This positioning solution is certainly an improvement from the SPP solution, which has an RMS of 2.0 m in the horizontal direction and 3.0 m in the vertical direction, in low-multipath and open-sky static environments.

Using developed measurement prediction and stochastic modeling approaches to condition the smartphone GNSS measurement, there is a 64% decrease in the horizontal positioning RMS error and 100% positioning solution availability with the car dashboard data collected in suburban environments. There is a 62% decrease in the positioning error and a 23% increase in positioning availability over regions where signals are significantly impacted by multipath and blockages. With the help of measurement prediction and C/N_0_-based stochastic modeling, the post-processed PPP solution had 100% availability and outperformed RTK in terms of availability of the solution. For the data processed, PPP is 84% more accurate than the internal smartphone solution, which suffers from biases due to non-constant tracking and has comparable accuracy with the RTK solution. With improvements in antenna quality and GNSS data loggers, the positioning accuracy of the smartphone-based PPP solution is expected to improve. Currently, only 14 GPS satellites transmit L5 frequency observations, which limits dual-frequency processing. Future work will involve further analysis, improvement and testing of the developed C/N_0_-based stochastic modeling and measurement prediction approaches, and for real-time PPP processing of smartphone measurements. The newly released second-generation, dual-frequency GNSS chips, such as the BCM47765, will also be tested. Lastly, the integration of the GNSS measurements with measurements from other smartphone sensors such as accelerometers and gyroscopes to obtain a tightly-coupled solution will be considered.

## Data Availability

The datasets used and analysed during the current study are available from the corresponding author on request.

## Author’s information

GS is an M.Sc. student in the Department of Earth and Space Science and Engineering at York University, Toronto, Canada. Previously, she completed her B.Tech. in Computer Science from Manipal Institute of Technology, India. Her research focus is PPP augmentation of smartphone GNSS. SB is a Professor in the Department of Earth and Space Science and Engineering at York University, Toronto, Canada. He received a Ph.D. in Geodesy and Geomatics Engineering from the University of New Brunswick. For over twenty-five years, he has been actively researching GNSS processing algorithms for positioning and navigation applications.
